# Supplementary material for: The Impact of Neurocognitive Functioning on the Course of Posttraumatic Stress Symptoms following Civilian Traumatic Brain Injury
Source: J Clin Med. 2021 Oct 30;10(21):5109. doi: 10.3390/jcm10215109 (PMC8584567; doi:10.3390/jcm10215109)
Supplement: Supplementary file 1 [file jcm-10-05109-s001.zip › jcm-1345273-supplementary.pdf]

Table S1. Cognitive covariates.

Test	Cognitive domain	Measures
Trail Making Test (TMT)		
TMT A	Attention and processing speed	Time (in secs) taken to draw lines between 25 numbers in sequence
TMT B	Attention, processing speed and task switching Cognitive flexibility	Time (in secs) taken to draw lines in alternating sequence between 25 letters and numbers
Rey Auditory Verbal Learning Test (RAVLT)		Number of words recalled from a list of 15 unrelated words read out by the assessor
RAVLT Immediate recall	Verbal short-term memory	Sum of the first 5 trials
RAVLT Interference recall	Interference	Trial 6 after an interference list
RAVLT Delayed recall	Verbal long-term memory	Trial 7 after 20 minutes
RAVLT Learning rate	Verbal learning	Trial 5 minus trial 1
RAVLT Forgetting rate	Consolidation verbal information	Trial 5 minus delayed recall (trial 7)
Cambridge Neuropsychological Test Automated Battery (CANTAB)		
CANTAB SWM: Spatial working memory	Spatial working memory	Find hidden tokens in displayed boxes. Outcome measure: the number of times a box is selected in which a token was already presented (number of errors)
CANTAB PAL: Paired associate learning	Visual learning and memory	Number of errors, adjusted for the estimated number of errors that could have been made on any problems, attempts and unfinished items
CANTAB RVP: Rapid visual information processing	Sustained attention and concentration	Detect specific sequences by pushing a button. Outcome measure: the accuracy of target detection calculated from hits and correct rejections

CANTAB SOC: Stockings of Cambridge task	Spatial planning and problem solving	Number of occasions upon which the participant successfully completes a test problem in the minimum possible number of moves
CANTAB RTI : Choice Reaction Time	Processing speed	Median duration (in secs) between the onset of the stimulus and the time at which the button is released
CANTAB AST: Attention Switching Task	Attention, task switching	Difference between the median latency of responses between assessments in the trial block in which the rule was switched vs those in the trial block in which the rule remained constant. Close to zero indicates less variation in latencies across non-switch and switch trials.

Table S2. Participant characteristics of the study sample and the participants with incomplete follow-up (FU) data.

Demographic characteristics	Study sample *n* = 671	Participants with incomplete FU *n* = 1401	*p*
Age (years)	49 (31–61)	50 (30–66)	0.021
> 65 years	102/671 (15.2)	357/1401 (25.5)	< 0.001
Male	463/671 (69.0)	959/1401 (68.5)	0.77
Highest educational level:			
College/University	188/614 (30.6)	268/1183 (22.7)	0.007
Married or living with partner	369/648 (56.9)	669/1401 (47.8)	<0.001
**Injury-related characteristics**			
Glasgow Coma Scale:			
Mild TBI (13–15)	474/671 (70.6)	1015/1401 (72.5)	0.72
Moderate TBI (9–12)	67/671 (10.0)	135/1401 (9.6)	
Severe TBI (3–8)	130/671 (19.4)	251/1401 (17.9)	
Cause of injury:			
Road traffic incident	321/656 (48.9)	527/1314 (40.1)	
Incidental fall	244/656 (37.2)	651/1314 (49.5)	<0.001
Violence /Assault /Mass violence	24/656 (3.7)	71/1314 (0.5)	
Suicide attempt	12/656 (1.8)	9/1314 (0.7)	
Care pathway:			
Admitted to hospital	320/671 (47.7)	731/1401 (52.2)	0.052
Intensive Care Unit	351/671 (52.3)	670/1401 (47.8)	
**Psychiatric history[1]**			
Depression	37/68 (54.4)	112/196 (57.1)	0.70
Anxiety	18/68 (26.5)	55/196 (28.1)	0.80
Sleep disorder	11/68 (16.2)	20/196 (10.2)	0.19
Substance abuse	8/68 (11.8)	53/196 (27.0)	0.010
**Psychiatric characteristics at follow up**			
PCL-5 [2] total score 6mo	8 (3–18)	8 (2–17)	0.28
PTSD probable diagnosis 6mo	79/671 (11.8)	70/804 (8.7)	0.052
PCL-5 total score 12mo	8 (2–18)	7 (2–15)	0.049
PTSD probable diagnosis 12mo	71/671 (10.6)	53/574 (9.2)	0.47
PHQ-9 [3] total score 6mo	3 (1–7)	3 (1–8)	0.46
GAD-7 [4] total score 6mo	2 (0–5)	2 (0–5)	0.20

Median, interquartile range and Mann-Whitney tests were used for age, PCL-5, PHQ-9, GAD-7. Frequency (*n*, %) and Pearson's Chi^2 test for the other variables. [1] Information on psychiatric history and type(s) of psychiatric disorder was obtained during the interview with the patient and/or caretaker upon hospital admission. [2] PCL-5 is Posttraumatic Stress Disorder Checklist-5, [3] PHQ-9 is Patient Health Questionnaire-9 and screens for depressive symptoms, [4] GAD-7 is Generalized Anxiety Disorder-7 and screens for anxiety symptoms.

Table S3. Descriptive statistics for the PCL-5 total scores at 6 and 12 months, differentiated for PTSD at 6 months.

	**Total sample** *n* = 671			**PTSD 6mo** *n* = 79			**No PTSD 6mo** *n* = 592					
	**Min**	**Max**	**Mean (SD)**	**Min**	**Max**	**Mean (SD)**	**Min**	**Max**	**Mean (SD)**	**t(df)**	**Hedges' g**	***p***
PCL-5 at 6 mo	0	68	12.74 (13.56)	19	68	41.01 (10.99)	0	50	8.97 (8.45)	-24.94 (90.71)	3.65	<0.001
PCL-5 at 12 mo	0	79	12.54 (13.89)	6	79	32.27 (16.87)	0	57	9.91 (11.05)	-11.46 (87.14)	1.88	<0.001

Independent-samples t-tests were conducted to compare PCL-5 scores in the PTSD and no-PTSD groups, assuming unequal variances. Effect sizes were calculated using Hedges' g because of the different sample sizes.

Table S4. Descriptive statistics for the PCL-5 cluster change scores differentiated for PTSD at 6 months.

		**PTSD 6mo** *n* = 79			**No PTSD 6mo** *n* = 592						
**Cluster**	**Range**	**Min**	**Max**	**Mean (SD)**	**Min**	**Max**	**Mean (SD)**	**t**	**df**	**Hedges' g**	***p***
Total	0–80	−20	45	−8.75 (14.06)	−41	35	0.94 (8.57)	5.98	85.89	1.03	<0.001
Intrusion	0–20	−12	11	−2.27 (4.69)	−8	17	0.26 (2.66)	4.69	84.80	0.85	<0.001
Avoidance	0–8	−6	5	−1.13 (2.31)	−6	8	0.14 (1.47)	4.76	86.60	0.80	<0.001
Cogn/Mood	0–28	−20	14	−2.92 (5.70)	−15	24	0.30 (4.16)	4.85	89.39	0.74	<0.001
Arousal	0–24	−14	8	−2.44 (4.67)	−11	15	0.23 (3.00)	4.95	86.75	0.82	<0.001

Independent-samples t-tests were conducted to compare PCL-5 change scores in the PTSD and no-PTSD groups, assuming unequal variances. Effect sizes were calculated using Hedges' g because of the different sample sizes.

Table S5. Linear Regression: Covariates Associated with PCL-5 Change Scores in Participants with a Probable PTSD Diagnosis (n = 79). .

Covariate	B	SE (B)	95% CI[1]		p[4]	VIF[5]
			LL[2]	UL[3]		
(Intercept)	12.61	8.34	−4.02	29.23	0.15	
Age	−0.15	0.11	−0.37	0.069	0.18	1.37
Psychiatric history	4.19	4.06	−3.91	12.28	0.31	1.06
GCS[6]	−0.81	.45	−1.71	0.09	0.078	1.23
CANTAB PAL[7]	6.31	1.77	2.78	9.84	0.001	1.70
RAVLT-Interference recall[8]	22.98	5.66	11.69	34.27	<0.001	1.79
GCS x RAVLT- Interference recall	−1.37	0.42	−2.20	−0.54	0.002	

[1] CI = confidence interval; [2] LL = lower limit; [3] UL = upper limit; [4] significance level $p < 0.01$; [5] VIF = variance inflation factor; [6] GCS = Glasgow Coma Scale; [7] CANTAB PAL = Cambridge Neuropsychological Test Automated Battery - Paired Associate Learning Test (high scores reflect weak visual learning and memory); [8] RAVLT Interference recall = Rey Auditory Verbal Learning Test – Interference recall (high scores reflect strong short-term memory), high PCL-5 change scores reflect worsening symptoms. The interaction effect for the PCL-5 change score and RAVLT-Interference recall was significant (RAVLT-Interference in mild TBI: B = 3.77, SE(B) = 2.00, p = 0.064, moderate TBI: B = 8.57, SE(B) = 2.15, p < 0.001, and severe TBI: B = 15.43, SE(B) = 3.59, p < 0.001).

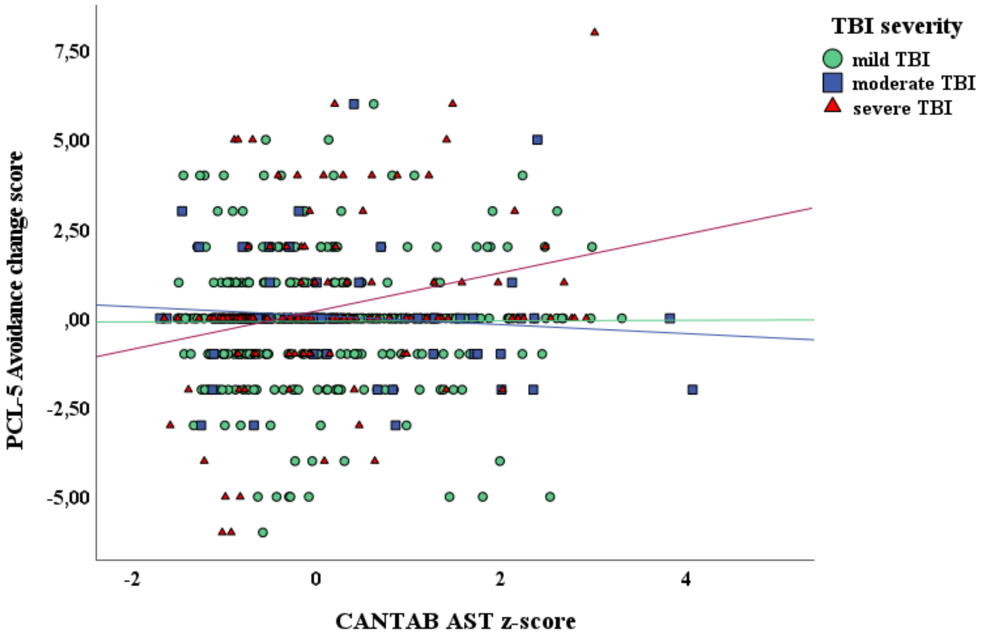

Figure S1. Scatterplot of the PCL-5 avoidance change scores and the CANTAB AST differentiated for Glasgow Coma Scale.

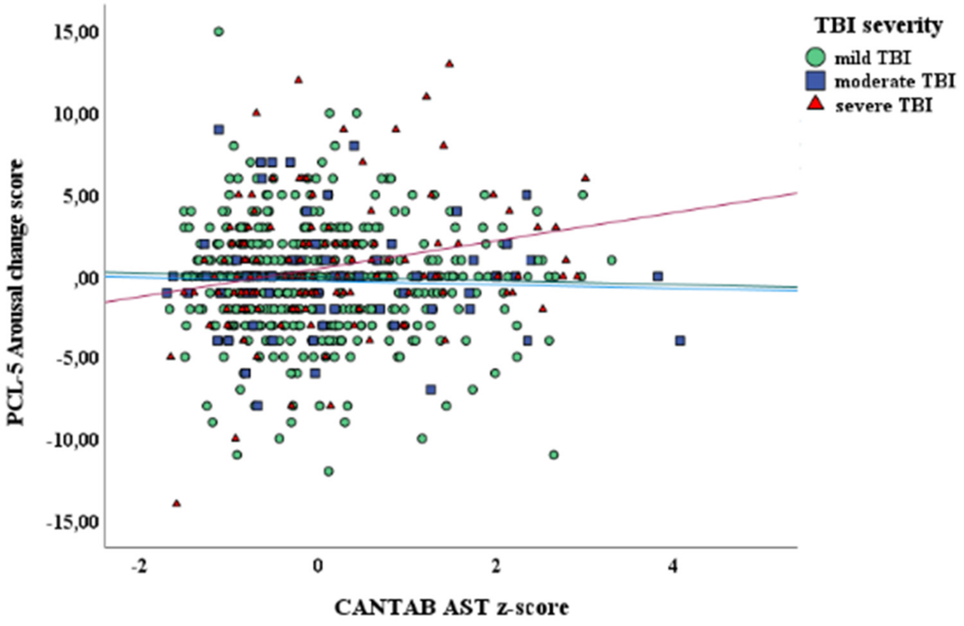

Figure S2. Scatterplot of the PCL-5 arousal change scores and the CANTAB AST differentiated for Glasgow Coma Scale.
